# Tomato Lycopene Extract Prevents Lipopolysaccharide-Induced NF-κB Signaling but Worsens Dextran Sulfate Sodium-Induced Colitis in NF-κB^EGFP^ Mice

**DOI:** 10.1371/journal.pone.0004562

**Published:** 2009-02-23

**Authors:** Young-Eun Joo, Thomas Karrasch, Marcus Mühlbauer, Brigitte Allard, Acharan Narula, Hans H. Herfarth, Christian Jobin

**Affiliations:** 1 Department of Medicine and Center for Gastrointestinal Biology and Disease, University of North Carolina at Chapel Hill, Chapel Hill, North Carolina, United States of America; 2 Narula Research, Chapel Hill, North Carolina, United States of America; 3 Department of Pharmacology, University of North Carolina at Chapel Hill, Chapel Hill, North Carolina, United States of America; Charité-Universitätsmedizin Berlin, Germany

## Abstract

**Background:**

The impact of tomato lycopene extract (TLE) on intestinal inflammation is currently unknown. We investigated the effect of TLE on lipopolysaccharide (LPS)-induced innate signaling and experimental colitis.

**Methodology/Principal Findings:**

Mice were fed a diet containing 0.5 and 2% TLE or isoflavone free control (AIN-76). The therapeutic efficacy of TLE diet was assessed using dextran sulfate sodium (DSS) exposed mice and IL-10^−/−^;NF-κB^EGFP^ mice, representing an acute and spontaneous chronic colitis model respectively. A mini-endoscope was used to determine the extent of macroscopic mucosal lesions. Murine splenocytes and intestinal epithelial cells were used to determine the *in vitro* impact of TLE on LPS-induced NF-κB signaling. *In vitro*, TLE blocked LPS-induced IκBα degradation, RelA translocation, NF-κB transcriptional activity and MIP-2 mRNA accumulation in IEC-18 cells. Moreover, LPS-induced IL-12p40 gene expression was dose-dependently inhibited in TLE-treated splenocytes. Interestingly, DSS-induced acute colitis worsened in TLE-fed NF-κB^EGFP^ mice compared to control diet as measured by weight loss, colonoscopic analysis and histological scores. In contrast, TLE-fed IL-10^−/−^;NF-κB^EGFP^ mice displayed decreased colonic EGFP expression compared to control diet. IL-6, TNFα, and MCP-1 mRNA expression were increased in the colon of TLE-fed, DSS-exposed NF-κB^EGFP^ mice compared to the control diet. Additionally, caspase-3 activation and TUNEL positive cells were enhanced in TLE diet-fed, DSS-exposed mice as compared to DSS control mice.

**Conclusions/ Significance:**

These results indicate that TLE prevents LPS-induced proinflammatory gene expression by blocking of NF-κB signaling, but aggravates DSS-induced colitis by enhancing epithelial cell apoptosis.

## Introduction

Lycopene is a phytochemical found in red fruits including apricot, papaya, watermelon and tomatoes. Dietary intake of tomatoes and tomato-based products has been associated with a reduced incidence of developing atherosclerosis, coronary heart disease and prostate cancer [1]–[4]. More than 80% of dietary lycopene intake is derived from raw and processed tomato products such as juice, spaghetti or pizza sauce [5]. For example, drinking three glasses (240 ml/glass) of a processed vegetable juice provides in excess of 40 mg of lycopene/day [6], a concentration associated with reducing LDL cholesterol [3]. The beneficial health properties of tomatoes have been attributed to the action of various compounds including dietary fiber, folates, vitamins and carotenoids. Among these, carotenoids have received much attention due to their ability to quench singlet oxygen and scavenge peroxyl radicals which is associated with the above mentioned health benefits [7]–[9].

Since lycopene is the major carotenoid present in tomatoes and their by-products, this phytochemical has been the focus of intense research to determine its impact of various disease states. Although primarily considered an antioxidant [10], [11], lycopene appears to have an influence on cellular proliferation and differentiation as well as immune response [12]. Interestingly, lipopolysaccharide (LPS)-induced phenotypic and functional maturation of murine dendritic cells is inhibited by lycopene both *in vitro* and *in vivo* [13]. In addition, lycopene decreased oxidative stress and intestinal inflammation in a rat model of experimental colitis [14]. These findings suggest that lycopene and/or tomato lycopene extract (TLE) possess anti-inflammatory properties, partially mediated through inhibition of innate host responses.

Crohn's disease and ulcerative colitis collectively referred to as inflammatory bowel disease (IBD) are chronic relapsing intestinal inflammatory disorders [15]. Although the etiology of IBD is currently unknown, converging evidence suggests that a pathological synergy exists between defective innate immune responses and uncontrolled lamina propria mononuclear (LPMNC) and T cell activation, playing a central role in disease pathogenesis [16]–[19]. Key to this dysregulated host response is the presence of intestinal microbiota, which in a genetically susceptible host, activates intestinal immune cells to release a number of inflammatory mediators such as IL-1, IL-6, IL-12p40, IL-23p19, TNFα and IFNγ [20]. A key transcription factor involved in the production of many of these inflammatory mediators is NF-κB [21]–[23]. We previously showed that pharmacological inhibition of NF-κB signaling prevents the development of bacteria-induced colitis in IL-10^−/−^ mice [24], [25].

In the present study, we examined the impact of TLE on LPS-induced innate signaling as well as acute and spontaneous chronic intestinal inflammation. We found that TLE prevents LPS-induced proinflammatory gene expression by blocking NF-κB signaling, through aggravation of DSS-induced colitis by enhancing epithelial cell apoptosis following injury.

## Materials and Methods

### Cell culture and treatment

The non-transformed rat small intestinal cell line IEC-18 (American Type Culture Collection (ATCC) CRL1589, Manassas, VA) was used between passages 25 and 40. Cells were cultured as described previously [26]. Spleens were collected from NF-κB^EGFP^ mice as described previously [27]. Primary colonic epithelial cells were isolated using Hank's balanced salt solution (Ca^2+^ and Mg^2+^ free, Invitrogen, Carlsbad, CA) containing 2 mmole/L EDTA as described previously [28]. TLE (Narula Research, Chapel Hill, NC, USA) was dissolved in dimethyl sulfoxide (DMSO; Sigma, St Louis, MO) to a final concentration of 50 mg/mL. Cells were pretreated with various concentrations of TLE (0–0.1 g/L) after which they were stimulated with LPS (5 mg/L; *Escherichia coli* serotype O111:B4, Sigma) or TNFα (10 µg/L or 40 µg/L; R & D Systems, Minneapolis, MN) for times indicated.

### Immunofluorescence

TLE (0.1 g/L) pretreated IEC-18 cells were stimulated with LPS (5 mg/L) for 1 h, fixed with 100% ice-cold methanol for 10 min at 4°C and RelA immunofluorescence was performed as described previously [29].

### NF-κB-luciferase reporter assay

IEC-18 cells were infected for 16 h with an adenoviral vector encoding a NF-κB-luciferase reporter gene (Ad5κB-LUC) as described previously [30]. Cells were then pretreated with various concentrations of TLE for 1 h, after which time, they were stimulated with LPS (5 mg/L) or TNFα (10 µg/L) for 12 h. NF-κB transcriptional activity was measured as described previously [27].

### Diet and colitis models

TLE-enriched diet was based on standard laboratory diet (AIN-76A) [31] by incorporating different amounts of 20% TLE (0.5% TLE, 28 g/kg diet; 2% TLE, 112 g/kg diet) (Research Diets Inc, New Brunswick, NJ). TLE composition is: Lycopene (∼20%), other carotenoid (5∼10%), flavonoids (0.5∼3%), protein (5∼10%), sugar (10∼20%) and tannin (15∼25%). For acute colitis studies, four groups of mice (n = 6) were exposed to 3% DSS (MP Biomedicals, Aurora, OH) in drinking water (group 1; positive control), drinking water alone (group 2; negative control), 0.5% TLE (group 3) and 2.0% TLE (group 4). Group 1–2 were fed AIN-76A (C) and group 3–4 were fed TLE for 4 d (loading period) before exposure to 3% DSS as described previously [28], [32]. Water consumption was comparable between the different groups. Consumption (C and TLE) was comparable between DSS and water control groups, both before and during induction of colitis (daily consumption approximately 2.5 g/mouse, equaling 12 mg TLE on a 2% diet). Mice were monitored daily for weight loss as well as signs of rectal bleeding and diarrhea. At d 4 of DSS administration, mice were sacrificed, sections were taken from the distal, proximal colon and cecum for histological assessment. EGFP expression was imaged as described previously [24], [28]. For spontaneous colitis, germ-free IL-10^wt/wt^;NF-κB^EGFP^ and IL-10^−/−^;NF-κB^EGFP^ littermates were transferred to a specific-pathogen-free (SPF) environment and immediately fed AIN-76A with or without 2%TLE. After 8 weeks, mice were euthanized, sections were taken from cecum, proximal and distal colon. Double blinded scoring was performed to evaluate severity of acute (DSS) and chronic colitis (IL-10^−/−^) in hematoxylin-eosin (H&E)-stained sections as described previously [24], [28]. For each animal, 2 sections approximately 400 µm apart were scored and averaged. All animal experiments were approved by the Institutional Animal Care and Use Committee of the University of North Carolina at Chapel Hill.

### Assessment of enhanced EGFP expression

NF-κB^EGFP^, IL-10^wt/wt^;NF-κB^EGFP^ and IL-10^−/−^;NF-κB^EGFP^ mice were sacrificed at the times indicated, the entire colon was dissected and then directly imaged for EGFP expression as described previously [24], [28].

### Immunohistochemical evaluation

Immunohistochemical staining for activated caspase 3 (Cell Signaling Technology Inc, Beverly, MA) was performed according to the manufacturer's directions and counterstained with Mayer's hematoxylin solution (Sigma).

### RNA extraction and amplification by RT-PCR

RNA was isolated using TRIzol (Invitrogen), reverse transcribed and amplified as previously described using primers specific for rMIP-2, mIL-12p40, mTNFα, mIL-6, mMCP-1, and mGAPDH. For PCR analysis, products were subjected to electrophoresis on 2% agarose gels containing GelStar fluorescent dye (Cambrex BioScience Rockland). Fluorescence staining was captured using an Alpha Imager 2000 (Alpha Innotech, San Leandro, CA). Cytokine expression was quantified using real-time PCR (Applied Biosystems 7900HT Fast Real-Time PCR System). Primer sequences were as follows; rat MIP-2, forward 5′-ACCCTACCAAGGGTTGACTTC-3′ and reverse 5′-GGCACATCAGGTACGATCCAG-3′; mIL-12p40, 5′-GAAGTTCAACATCAAGAGCAGTAG-3′ and 5′-AGGGAGAAGTAGGAATGGGG-3′; mTNFα, 5′-ATGAGCACAGAAAGCATGATC-3′ and 5′-TACAGGCTTGTCACTCGAATT-3′; mIL-6, 5′-CGGAGGCTTGGTTACACATGTT-3′ and 5′-CTGGCTTTGTCTTTCTTGTTATC-3′; mMCP-1, 5′-CCCAGCCAGATGCAGTTAACGCCCCACT-3′ and 5′-TTCACTGTCACACTGGTCACTC-3′; mGAPDH, 5′- GGTGAAGGTCGGTGTGAACGGA-3′ and 5′- GTGGGGTCTCGCTCCTGGAAGA –3′.

### Western blot analysis

Proteins were separated using SDS-PAGE and transferred to nitrocellulose membranes. Antibodies to cleaved caspase-3 and β-actin (ICN; Costa Mesa, CA) were diluted 1∶1000 in 0.1% TBS-Tween with 5% milk. Immunoreactive proteins were detected using the enhanced chemiluminescence light (ECL) detecting kit (Amersham Biosciences, Piscataway, NJ) as described previously [26].

### Cytokine measurement

Splenocytes were stimulated for 24 h with LPS (5 mg/L), supernatants collected, and cytokine levels measured using ELISA specific for IL-12p40 (BD pharmingen) according to the manufacturer's instructions.

### Colonoscopy

Endoscopy was performed on 4 d after the start of DSS administration. Direct visualization of the colon *in vivo* was performed using a “Coloview system” (Karl Storz Veterinary Endoscopy). Mice were supplied with food and water until the endoscopy was performed. If fecal material obstructed the view of the endoscope, colons were flushed with 0.9% saline. For the colonoscopies, the mice were anesthetized with 1.5 to 2% isoflurane and 3 to 4 cm of the colon from the anal verge until the splenic flexure was visualized after inflation of the colon with air. The colonoscopic procedures were digitally recorded on an AIDA Compaq PC.

### Assessment of Apoptosis

Mice were sacrificed d 3 after DSS-exposure and before loss of crypt structure and surface epithelium was observed. Apoptosis was evaluated by terminal deoxynucleotidyl transferase–mediated deoxyuridine triphosphate nick-end labeling (TUNEL) assay (DeadEnd™ Fluorometric TUNEL System; Promega, Madison, WI).

### Statistical analysis

Data are expressed as means±SEM. Groups of data (histological scores, body weight) were analyzed using Kruskal-Wallis non-parametric test (ANOVA) and the Mann-Whitney U test. For in vitro experiments, data were analyzed using a paired Student's *t* test and differences were considered significant if 2-tailed *p* values were <0.05.

## Results

### TLE inhibits LPS-induced NF-κB signaling and gene expression

**Figure 1:**
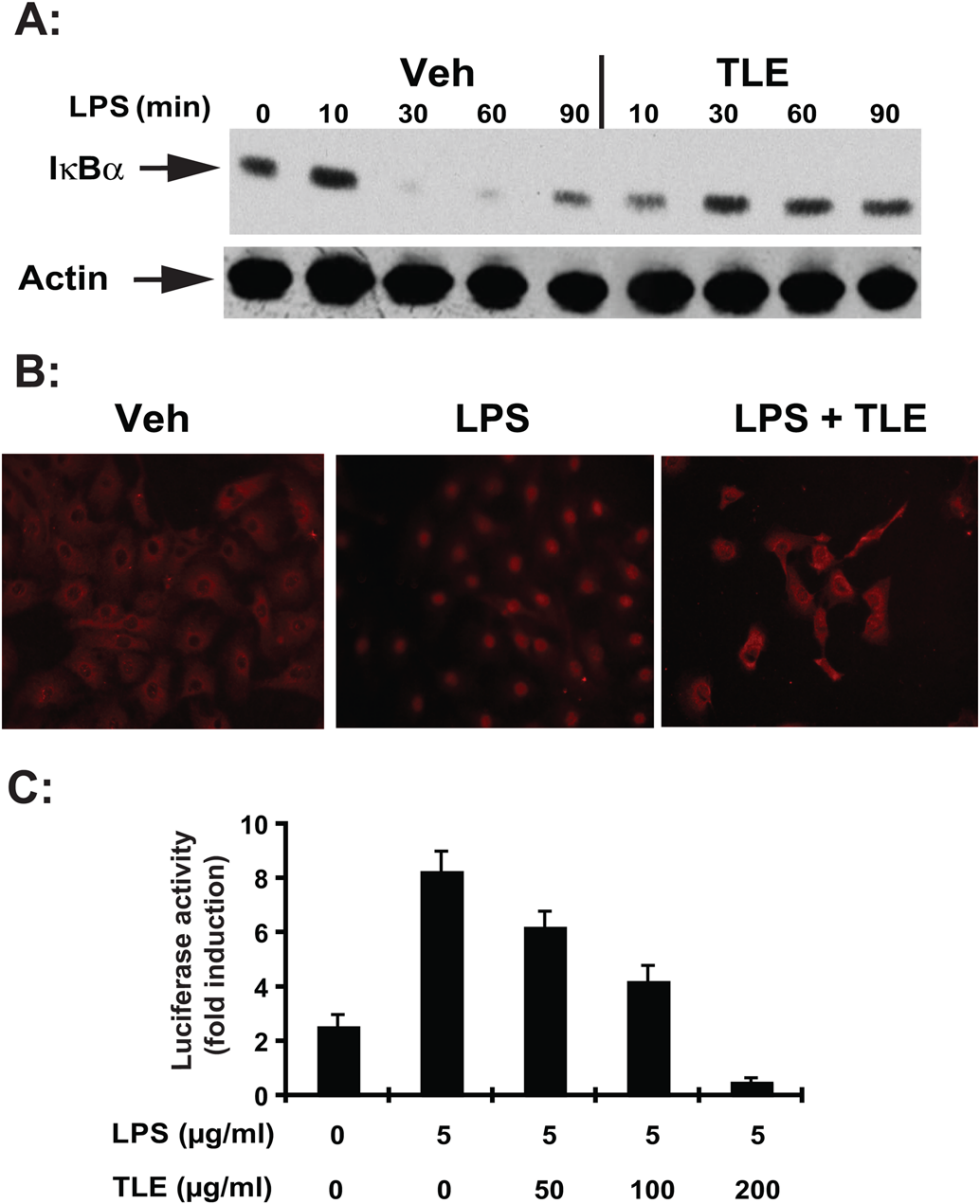
TLE inhibits LPS-induced NF-κB signaling in IEC-18 cells. (A) IκBα protein level was determined by Western blot analysis. β-actin was used as a loading control. Representative results of 3 independent experiments are shown. (B) Impact of TLE on LPS-induced RelA nuclear translocation was visualized using immunofluorescence staining. Representative results of two independent experiments are shown. (C) The effect of TLE on LPS-induced NF-κB transcriptional activity was assayed using an NF-κB-luciferase reporter gene (Ad5κB-LUC). Luciferase activity is expressed as relative units determined as the mean±SEM of three independent experiments measured in triplicate. Data were analyzed by the Student's *t* test. Experiments were performed in triplicate and repeated three times. Veh, vehicle.

**Figure 2:**
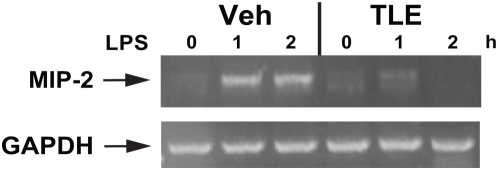
TLE inhibits LPS-induced MIP-2 mRNA expression in IEC-18 cells. The IEC-18 cells were pretreated with TLE (100 µg/mL) for 1 and 2 hr, stimulated with LPS (5 µg/mL) for various times. RNA was isolated using TRIzol procedure, and 1 µg of total RNA was reverse transcribed and amplified using specific primer for MIP-2 and GAPDH. Results are representative of 3 independent experiments.

**Figure 3:**
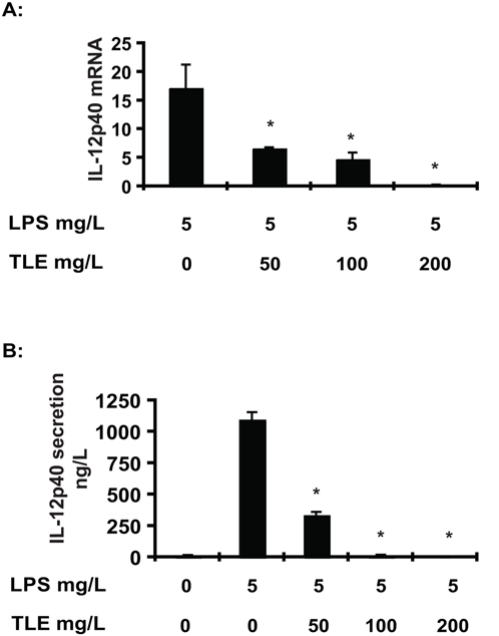
TLE inhibits LPS-induced IL-12p40 gene expression in murine splenocytes. (A) LPS-induced IL-12p40 mRNA accumulation following TLE treatment and LPS stimulation was measured using an ABI 7900HT Fast Real-Time PCR System. GAPDH was used as an internal control. Data are expressed as fold of the control (B) IL-12p40 secretion was measured by ELISA. Values are mean±SEM. All data were analyzed by Student's *t* test. Experiments were performed in triplicate and repeated three times.

LPS-induced IκBα degradation was inhibited in TLE-treated cells (Fig. 1A) which correlated with reduced LPS-induced RelA nuclear translocation (Fig. 1B). In addition, TLE dose dependently inhibited LPS-induced NF-κB transcriptional activity in IEC-18 cells (Fig. 1C). These effects were not specific to LPS since TNFα-induced RelA nuclear translocation and NF-κB transcriptional activity were also blocked in TLE-treated cells (data not shown). Furthermore, LPS-induced MIP-2 mRNA accumulation was inhibited by TLE treatment (Fig. 2). TLE dose-dependently inhibited LPS-induced IL-12p40 mRNA (Fig. 3A) and protein secretion (Fig. 3B) in splenocytes, indicating that the action of this dietary compound is not limited to IEC.

### TLE-treatment worsens acute but not chronic intestinal inflammation

**Figure 4:**
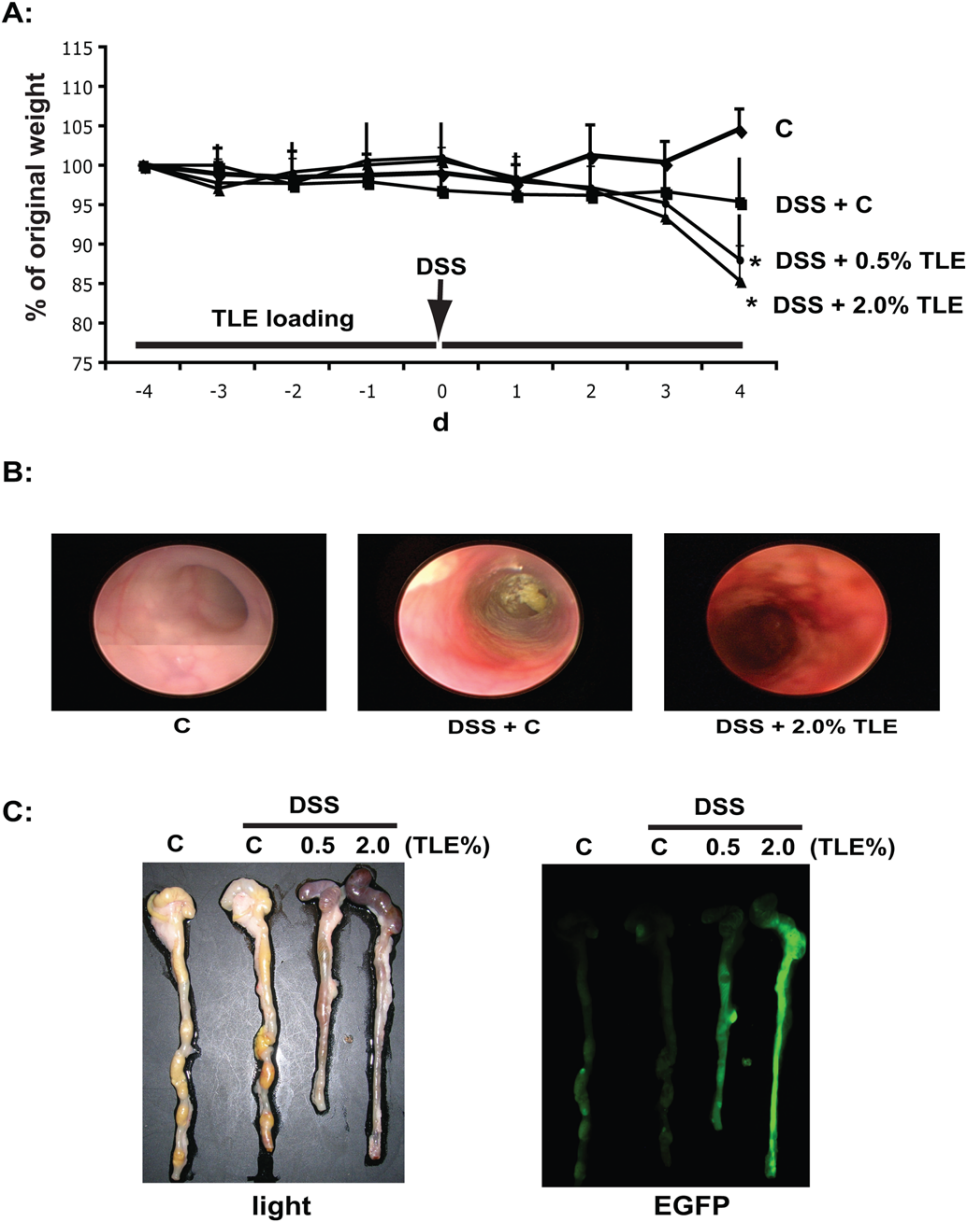
TLE worsens DSS-induced experimental colitis. (A) Weight lost in TLE or control diet-fed mice exposed to 3% DSS. Values are mean±SEM; n = 6 in each group. Data were analyzed by ANOVA and comparison between groups performed using the Mann-Whitney U test. Results are representative of 3 independent experiments. (B). Colonic inflammation visualized by colonoscopy after 4 d of 3% DSS-exposure. Representative images of 6 different mice are shown. (C) The Colons of NF-κB^EGFP^ mice were imaged using an EGFP macroimaging system. Photomicrographs are shown on the left. Representative images of 6 different mice are shown. C, AIN76A diet.

**Figure 5:**
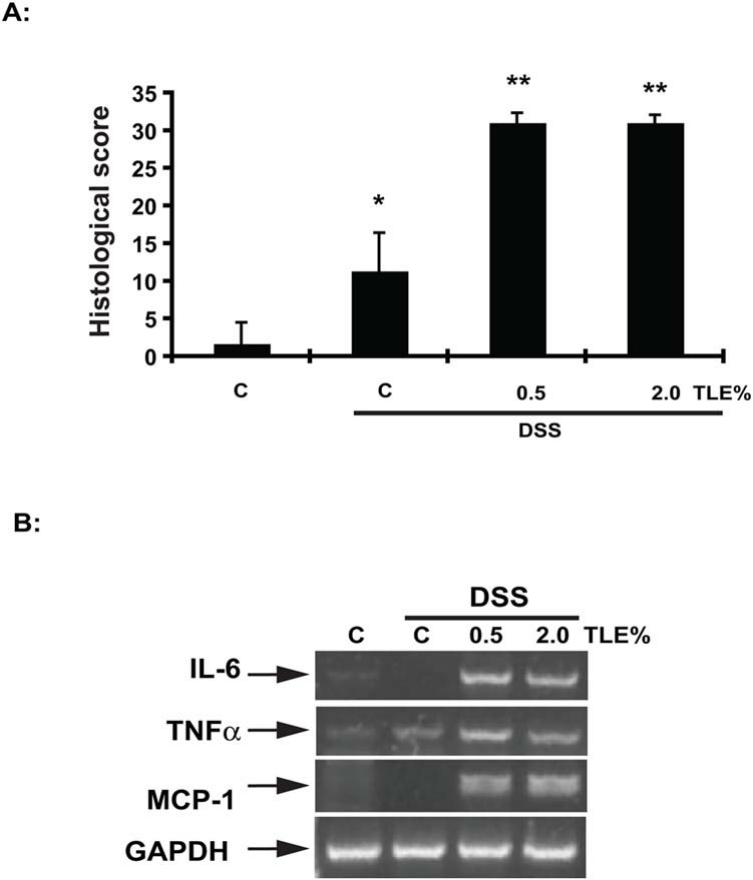
TLE exacerbates DSS-induced histological colitis and increased inflammatory gene expression. (A) Colonic histological sections were scored as described in Materials and Methods. Data were analyzed by ANOVA and comparison between groups performed using the Mann-Whitney U test. Values are mean±SEM; n = 6 in each group. Results are representative of 3 independent experiments. (B) IL-6, TNFα and MCP-1 mRNA accumulation from total colonic samples was determined using RT-PCR. GAPDH was used as an internal control. Representative results of 3 independent experiments are shown. C, AIN76A diet.

**Figure 6:**
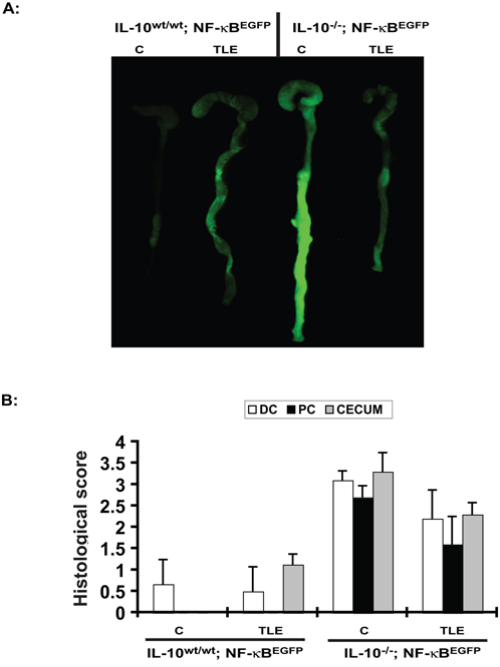
TLE diet decreases colonic EGFP expression in SPF-associated IL-10−/−;NF-κBEGFP mice. (A) The colons of TLE- or C diet fed IL-10^wt/wt^; NF-κB^EGFP^ mice and IL-10^−/−^;NF-κB^EGFP^ mice (4 weeks) were imaged using an EGFP macroimaging system. Representative images of 6 different mice are shown. (B) Histological damage from the distal and proximal colon (DC; PC) and the cecum from SPF-associated IL-10^wt/wt^; NF-κB^EGFP^ mice and IL-10^−/−^;NF-κB^EGFP^ mice. Data were analyzed by ANOVA and comparison between groups (C versus TLE; IL-10^−/−^ mice) performed using the Mann-Whitney U test. Values are mean±SEM; n = 6 in each group. C, AIN76A diet.

To test the therapeutic potential of TLE on intestinal inflammation, NF-κB^EGFP^ mice were pre-fed TLE or AIN-76A (control; C) and then exposed to 3% DSS. Surprisingly, by d 4 TLE-fed mice showed enhanced weight loss compared to control diet-fed mice (Fig. 4A). Colonoscopic analysis demonstrated that TLE-fed mice exhibited increased colonic inflammation with prominent mucosal edema and spontaneous bleeding compared to DSS-exposed mice on the control diet (Fig. 4B). Accordingly, the colon of TLE-fed, DSS-exposed NF-κB^EGFP^ mice displayed enhanced EGFP expression compared to control diet-fed mice (Fig. 4C). Histological evaluation of the colon of TLE-fed, DSS-exposed NF-κB^EGFP^ mice revealed an increase in colonic inflammation compared to DSS-exposed, control diet-fed mice (*P*<0.05) (Fig. 5A). Colonic expression of IL-6, TNFα and MCP-1 mRNA were elevated in TLE-fed mice compared to control diet mice (Fig. 5B). We next tested whether TLE similarly exacerbates intestinal inflammation using the spontaneous Th1-mediated IL-10^−/−^ model. Interestingly, TLE diet attenuated bacteria-induced colonic EGFP expression in IL-10^−/−^;NF-κB^EGFP^ mice compared to control diet (Fig. 6A). In addition, histological score indicates a decrease in distal, proximal and cecal inflammation (Fig. 6B). These results showed that TLE do not increase NF-κB activity (EGFP expression) nor exacerbate colitis in this spontaneous model.

### Impact of TLE on intestinal epithelial cell apoptosis

**Figure 7:**
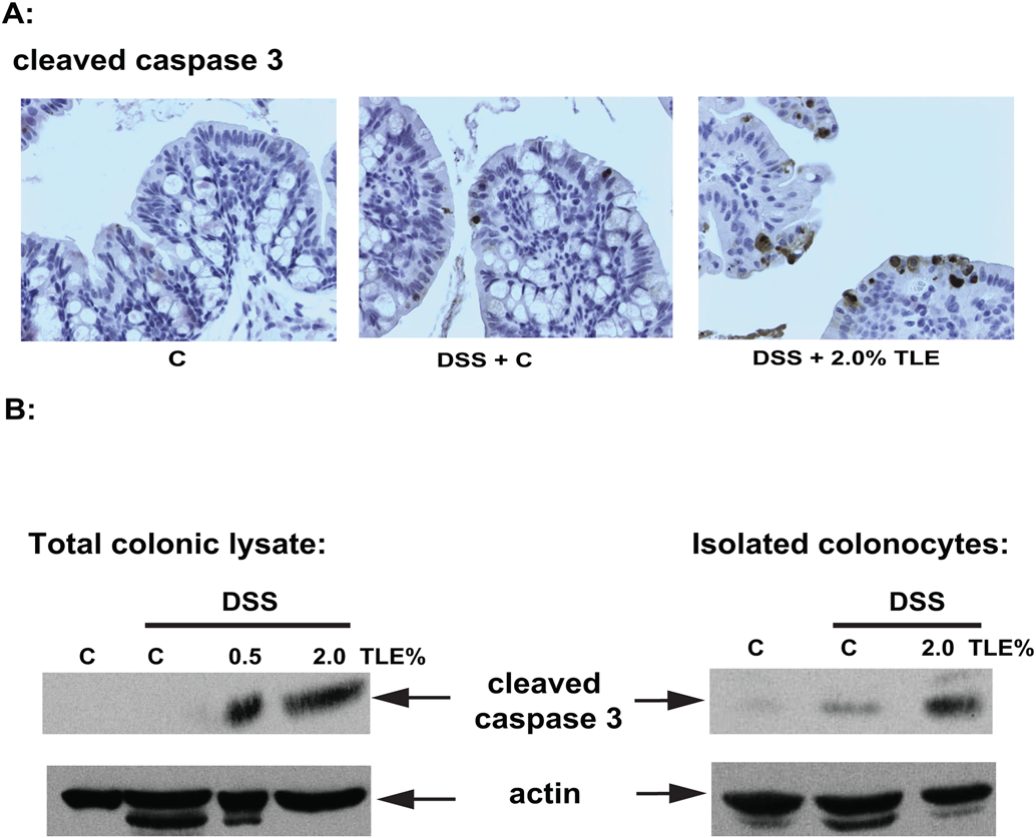
Enhanced caspase 3 processing in TLE-fed, DSS exposed mice. A) Cellular localization of cleaved caspase-3 was analyzed in the colon using immunohistochemistry (original magnification 200×). Representative results of 3 independent experiments are shown. (B) Caspase 3 processing in total colonic lysates (upper panel) and isolated colonocytes (lower panel) was determined by Western blot analysis. β-actin was used as a loading control. Representative results of 3 independent experiments are shown. C, AIN76A diet.

**Figure 8:**
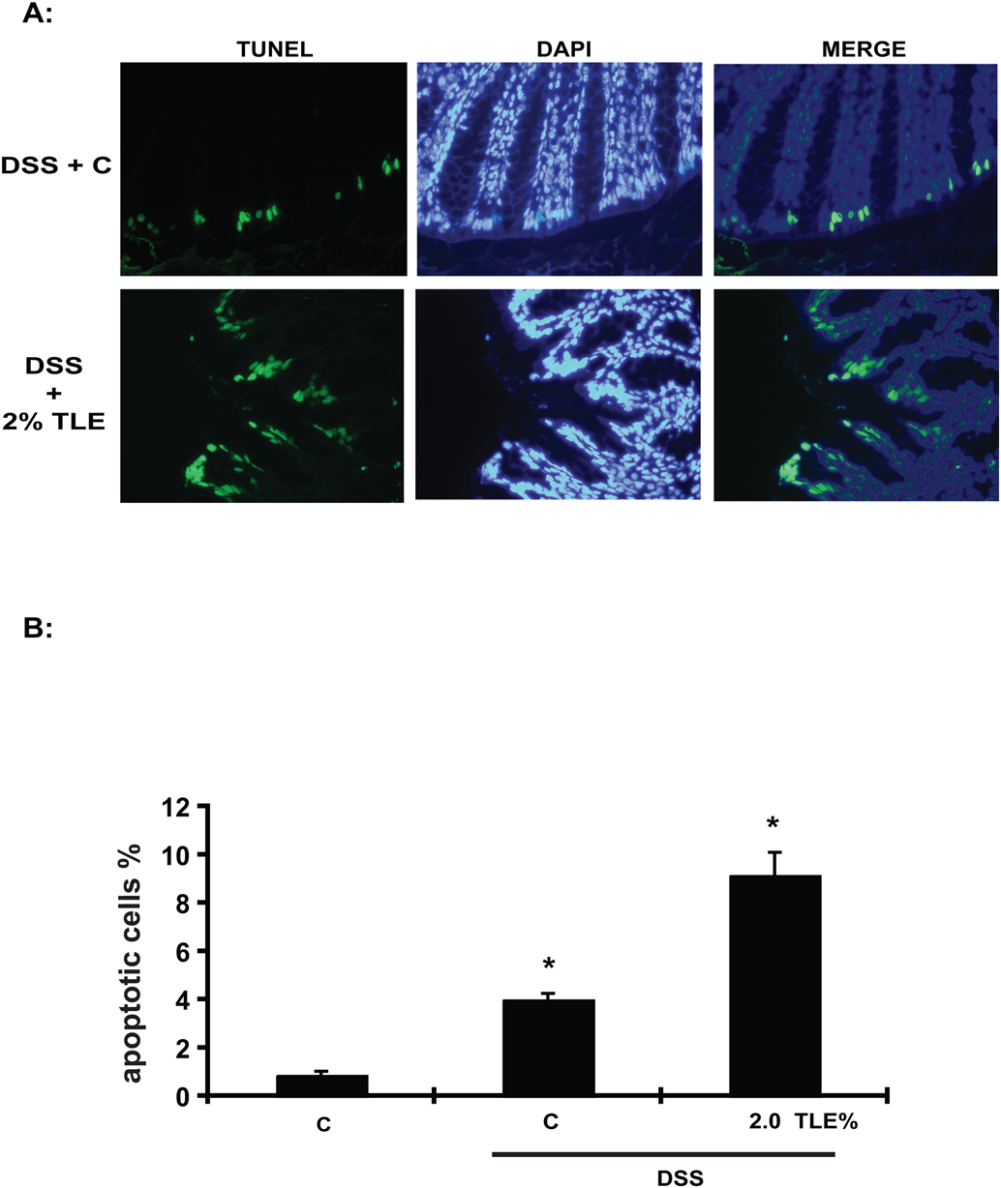
Increased apoptosis in TLE-fed, DSS-exposed mice. (A) Numbers of TUNEL positive cells in C, DSS+C and DSS+TLE (n = 3/group). Bars show the mean±SEM of the number of apoptotic cells per 100 total nuclei. Data were analyzed by ANOVA and comparison between groups performed using the Mann-Whitney U test. (B) Apoptotic cells in colon crypt epithelial cells of TLE-fed and control diet-fed mice were determined by fluorometric TUNEL assay. Representative sections were taken on 3 d of DSS-exposure. Representative results of 3 independent experiments are shown. C, AIN76A diet.

**Figure 9:**
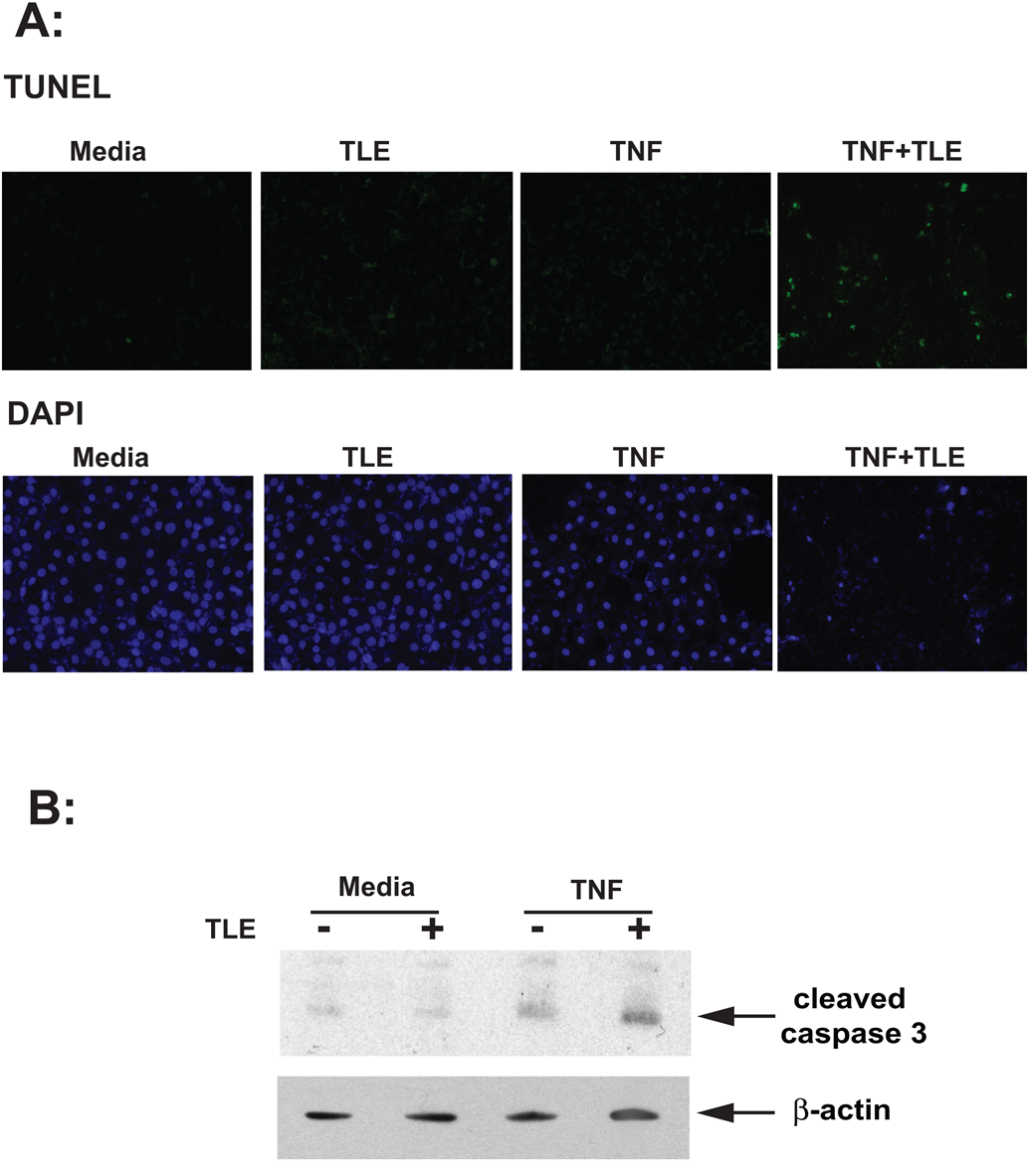
Increased apoptosis in TLE-treated, TNF-stimulated IEC-18 cells. (A) Apoptotic cells in IEC-18 cells pretreated with TLE (100 µg/ml; 45 min) and stimulated with TNFα (40 ng/ml) for 12 h were determined by fluorometric TUNEL assay. Representative field of 3 independent experiments are shown. (B) Caspase 3 processing in IEC-18 cells stimulated with TNFα (40 ng/ml) in presence or absence of TLE (100 µg/ml) was determined by Western blot analysis. β-actin was used as a loading control. Representative results of 3 independent experiments are shown.

A key feature of intestinal homeostasis is the host's ability to maintain the integrity of the epithelium and promote repair mechanisms following various injury. Therefore, we reasoned that TLE-fed mice could have increased IEC apoptosis in response to DSS-induced injury due to impaired NF-κB activity. Thus, we next sought to investigate the impact of TLE on intestinal apoptosis following DSS-induced injury. Since molecular alterations likely precede clinical signs of colitis and histopathological evidence of inflammation, (which occurred at around d 4–5 in our model) signs of apoptosis were evaluated 3 d after the start of DSS. Interestingly, immunohistochemical analysis demonstrated a strong increase of activated caspase-3 positive cells in TLE-fed, DSS-exposed mice compared to DSS-exposed mice (Fig. 7A). Moreover, western blot analysis performed on either isolated intestinal epithelial cells or total colon samples confirmed an increase in caspase-3 processing in TLE-fed, DSS-exposed mice compared to those isolated from DSS control mice (Fig. 7B). In accordance with the caspase-3 staining data, an increase in TUNEL positive cells was observed in TLE-fed, DSS-exposed mice compared to DSS-exposed control mice (*P*<0.05) (Fig. 8A). In general, the apoptotic cells were located in the surface epithelium (Fig. 8B). Moreover, in vitro experiments using IEC-18 cells showed that TLE enhanced TNF-induced apoptosis as measured by TUNEL staining (Fig. 9A) and caspase 3 processing (Fig. 9B). These findings indicate that TLE prevents NF-κB activity and exacerbates acute intestinal inflammation through enhanced epithelial apoptosis and reduced barrier function following injury.

## Discussion

In this study, we investigated the impact of TLE on LPS signaling in vitro and in experimental colitis. TLE prevented LPS-induced NF-κB activity both in IEC-18 cells and splenocytes through blockade of IκBα degradation, RelA nuclear translocation and transcriptional activity. TLE comprised of numerous components including vitamin C, polyphenols and carotenoids. Though our study has not directly identified the active ingredients responsible for this effect, TLE-mediated inhibition of NF-κB activity was replicated using pure lycopene (data not shown). Therefore this carotenoid is at least partially responsible for this inhibitory activity.

Despite its inhibitory effect in vitro, DSS-induced acute colitis was exacerbated in TLE fed NF-κB^EGFP^ mice. Mice fed with a TLE rich diet and exposed to DSS lost more weight than mice fed the control diet. This weight loss was not observed in non DSS treated fed TLE, indicating that the diet alone is not responsible for the phenotype. Fluorescent macro-imaging of the colon of TLE-fed, DSS-exposed NF-κB^EGFP^ mice showed a strong increase in EGFP expression compared to control diet mice, indicating enhanced NF-κB activity in these mice. Of note, EGFP expression was not increased in control diet-fed, DSS-exposed mice at d 4, which correlated with minimal weight loss. In contrast, increased EGFP expression in TLE-fed, DSS-exposed NF-κB^EGFP^ mice correlated with pronounced weight loss and histological evidence of intestinal damage. Moreover, increased levels of the NF-κB dependent genes IL-6, TNFα and MCP-1 were detected in TLE-fed, DSS-exposed NF-κB^EGFP^ mice. Consequently, exacerbated colitis correlated with enhanced NF-κB activation and pro-inflammatory gene expression in TLE-fed, DSS-exposed NF-κB^EGFP^ mice. The 2% diet utilized in this study corresponds to 12 mg lycopene/day. This amount falls within the range comsumed in a typical human diet, with for example, spaghetti sauce (21 mg/serving), watermelon (11 mg/serving), tomatoes (14 mg/serving) and tomato juice (19 mg/serving) providing from 10–40 mg of the phytochemical [6].

The deleterious effect of TLE on DSS-induced colitis is likely specific to the model on intestinal injury. Indeed bacteria-induced EGFP expression was reduced in TLE-fed IL-10^−/−^;NF-κB^EGFP^ mice compared to control diet fed mice, with a concomitant attenuation of cecal and proximal colonic inflammation. These findings suggest that the deleterious effects of TLE relates to the injury-based mechanism of the DSS model, which cause disruption of the epithelial cell layer resulting in breakdown of intestinal barrier function. This breach of barrier function would likely result in increased uptake of luminal antigens (bacteria and bacterial products) as well as activation of lamina propria immune cells and inflammatory response [18]–[20]. This hypothesis reconciles the apparent discordance between enhanced NF-κB activation in the colon of TLE-fed, DSS exposed mice and the inhibitory action of the phytochemical in vitro and in the IL-10^−/−^ model.

NF-κB drives expression of target genes that function to protect IEC from signal-induced apoptosis as well as promoting restitution of the epithelium [18]. A potential explanation for exacerbated colitis in TLE-treated, DSS-exposed mice may be that TLE impair barrier function thereby increasing the susceptibility of IEC to undergo apoptosis. This possibility is supported by our immunohistochemical and western blot analysis showing that the colon of TLE-fed mice displayed enhanced caspase-3 processing. Increased caspase-3 processing has been previously associated with DSS-induced colonic tissue damage and colitis [33]. Furthermore, higher number of TUNEL positive IEC were observed in TLE-fed, DSS-exposed mice. In contrast, the number of TUNEL positive cells was similar between control diet and TLE fed IL-10^−/−^;NF-κB^EGFP^ mice (data not shown). In addition, TNF-induced apoptosis increased in IEC-18 cells exposed to TLE. These findings support the notion that TLE interferes with NF-κB-mediated pro-survival signals leading to increased apoptosis in the injured epithelium, thereby compromising barrier integrity. Since irradiation therapy, bacterial infection and non-steroidal anti-inflammatory drug exposure is associated with intestinal injury, a diet rich in lycopene could potentially interfere with repair mechanisms implicated in the restoration of the epithelium.

## Acknowledgments

We thank Maureen Bower and Silmara Camargo at the Gnotobiotic Core Facility of the Center for Gastrointestinal Biology and Disease for their expert help in germ-free rodent technology. The authors thank Kathy Thompson from the Histology Core and Rosemary Link from the Immunoassay Core of the Center for Gastroenterology Biology and Disease for expert assistance in H&E histology and Il-12p40 quantification, respectively. We thank Dr. Joseph A. Galanko in the Center for Gastrointestinal Biology and Disease at the University of North Carolina at Chapel Hill for his expert help on statistical analyses. The authors wish to thank Dr. Josh Uronis for his critical review of the manuscript.
